# Comparison of outcome of patients with CLL who are referred or nonreferred to a specialized CLL clinic: a Canadian population‐based study

**DOI:** 10.1002/cam4.559

**Published:** 2016-02-18

**Authors:** Sara Beiggi, Versha Banerji, Angela Deneka, Jane Griffith, Spencer B. Gibson, James B. Johnston

**Affiliations:** ^1^ Department of Biochemistry and Medical GeneticsUniversity of Manitoba745 Bannatyne AvenueWinnipegManitobaR3E 0J9Canada; ^2^ Manitoba Institute of Cell BiologyCancer Care Manitoba675 McDermot AveWinnipegManitobaR3E 0V9Canada; ^3^ Department of Internal MedicineUniversity of Manitoba820 Sherbrook StreetWinnipegManitobaR3E 2N2Canada; ^4^ Department of Hematology and Medical OncologyCancer Care Manitoba675 McDermot AveWinnipegManitobaR3E 0V9Canada; ^5^ Department of Epidemiology and Cancer RegistryCancer Care Manitoba675 McDermot AveWinnipegManitoba R3E 0V9Canada; ^6^ Department of Community Health SciencesUniversity of Manitoba750 Bannatyne AvenueWinnipegManitoba R3E 0W3Canada

**Keywords:** Chronic lymphocytic leukemia, CLL clinic, outcome, overall survival, referral, small lymphocytic lymphoma

## Abstract

Chronic lymphocytic leukemia and small lymphocytic lymphoma (CLL/SLL) patients in Manitoba are either referred to the CLL Clinic at CancerCare Manitoba (CCMB) or are followed by other hematologists and general practitioners. However, it has been unclear whether referral to the CLL clinic influences patient outcome. Overall survival (OS) was assessed for all CLL/SLL patients diagnosed in Manitoba between 2007 and 2011. Of 555 patients, 281 (51%) were referred to the CLL clinic. Patients seen in this clinic had a twofold increased OS compared to patients who were managed by other hematologists and general practitioners (HR 2.375, *P* 0.0002) when adjusted for age, gender, presence of pre‐ or post‐CLL cancer, treatment and urban/rural location. In the nonreferred population there was a striking correlation between advancing age and decreasing OS. However, this correlation was almost eliminated in the referred population who were more likely to receive chemotherapy. Patients referred and seen in the CLL clinic have an improved OS compared to nonreferred patients and this appears to be primarily related to improved OS in the elderly. Possible explanations for this finding are discussed.

## Introduction

Chronic lymphocytic leukemia/small lymphocytic lymphoma (subsequently referred to as CLL) is the most common leukemia in the western world with an incidence rate of 7.9/100,000 persons in Manitoba 1. Over the past 20 years, the relative survival of CLL patients (calculated by correcting for the survival of age‐ and sex‐matched control population) both in epidemiological studies and in CLL clinics has significantly improved, except for those aged >70–80 years 1, 2. The increased relative survival in younger patients may be related to improved therapies and supportive care, but it remains unclear why the relative survival in the elderly has not improved 2, 3, 4.

In Manitoba, CLL patients are either referred to a specialized CLL Clinic at CancerCare Manitoba (CCMB) or are cared for by other hematologists or by their family practitioners. To date, it is unknown whether referral to the CLL clinic in Manitoba has affected patient outcome. The concept that clinical outcome could be improved by treatment in a high volume center was first introduced more than three decades ago 5. Since then, it has been shown in the literature that a physician's subspecialty and caseload as well as center volume are directly associated with a more favorable outcome for nonsurgical patients 6, 7, 8, 9. Furthermore, it has been demonstrated that newly diagnosed CLL patients who are followed by CLL specialists at the Mayo Clinic have a longer time to first treatment (TTFT) and better overall survival (OS) compared with patients seen by other hematologists within the same center 10.

In this study, we used Manitoba Cancer Registry and immunophenotypic data to create a population‐based cohort of CLL patients in the province of Manitoba, Canada, identified over a 5‐year period (2007–2011). We evaluated the OS of these patients to determine whether there was a difference between patients referred to a specialized CLL clinic at CCMB compared to patients who were managed by other hematologists and/or family doctors.

## Methods

### Patients

All cases diagnosed with CLL and SLL between January 1, 2007 and December 31, 2011 were obtained from the Manitoba Cancer Registry. In Manitoba, flow cytometry is performed centrally, and all reports demonstrating the presence of CLL cells (monoclonal B lymphocytes that are CD19^+^, CD5^+^, and CD23^+^) are reported to the Provincial Cancer Registry by provincial law. Other cancers diagnosed within 30 days prior or subsequent to the CLL diagnosis were considered concurrent malignancies. Data pertaining to causes of death was collected from death certificates through the Provincial Cancer Registry. Rural patients are defined as patients residing outside the city of Winnipeg as determined by postal codes (Brandon is the largest city in Manitoba after Winnipeg with a population of approximately 46,000). Patients referred to the CLL clinic at CCMB were identified from CCMB medical records. In the CCMB clinic, the 2008 updated iwCLL guidelines for diagnosis and treatment of CLL were used 11, and accordingly patients with monoclonal B‐cell lymphocytosis (MBL) were eliminated from the referred patient cohort; however, it is likely that the nonreferred cohort included some MBL patients. Patients who had been initially followed by general hematologists and family physicians but later referred to the CLL clinic were included in the referred cohort. Data pertaining to the types of first treatment for referred patients were obtained from CCMB patient records. Ethics approval was obtained from the University of Manitoba Health Research Ethics Board.

### Statistical analysis

Time to first treatment was calculated from the date of CLL diagnosis to the date of administration of the first treatment. OS was calculated as the time between date of diagnosis and date of death or end of study (December 31, 2011). The associations between age, referral status and treatment were measured by chi‐squared test. Univariable analysis was performed using Kaplan‐Meier methods. The Cox Proportional Hazard Regression model was used to estimate Hazard Ratios (HRs) in univariable and multivariable models. A *P*‐value <0.05 was considered to be statistically significant. Data management and analysis was performed using Microsoft Excel (Microsoft Corp., Redmond, WA) and SAS 9.2 (SAS Institute Inc., Cary, NC).

## Results

### Patient population

**Figure 1:**
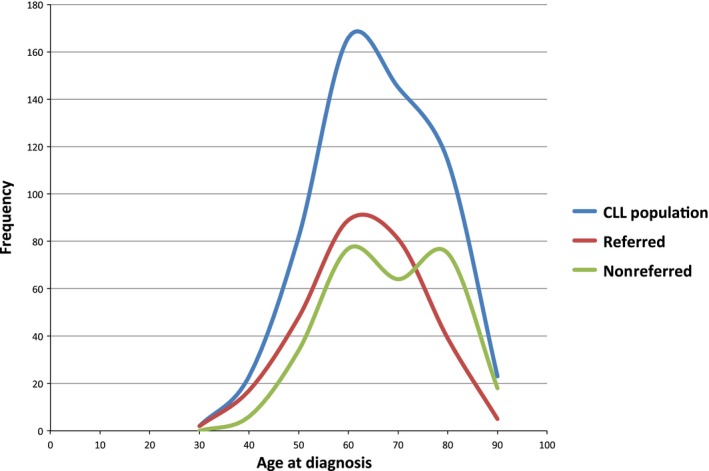
Age distribution of manitoba chronic lymphocytic leukemia population.

There were 555 CLL diagnoses made in Manitoba between 2007 and 2011. Of the 555 patients, 281 (51%) were referred to the CCMB specialized CLL clinic (referred patients) and 274 (49%) were seen by other hematologists (193 cases or 70% of nonreferred patients) or followed by their family practitioners (81 cases or 30% of nonreferred patients), both combined and termed nonreferred patients. The clinical features of these two groups of patients are shown in Table 1. Patients referred to the clinic were younger than nonreferred patients (*P* = 0.0033) and the median age of referred patients was 68 years compared to 72 years for nonreferred patients. In addition, while the age for referred patients was in a normal distribution with a peak at 60 years, age for nonreferred patients had a bimodal distribution with peaks at approximately 60 and 80 years (Fig. 1). The primary causes of death in both referred and nonreferred cohorts were CLL, the complications of CLL or second malignancies. Comorbidities such as cardiovascular diseases had equal contributions to mortality in both populations (7% and 9.5% in referred and nonreferred patients, respectively) (Table 2). When patients older than 70 years were further analyzed, elderly patients who were referred to the clinic were more likely to receive treatment (*P* = 0.0002). The most common treatment regimens in the clinic were fludarabine/rituximab combinations (36%) or single‐agent chemotherapy (41%), including chlorambucil, fludarabine, or cyclophosphamide. Twenty‐three percent of patients received initial prednisone, either for immune cytopenia or to clear marrow before chlorambucil.

**Table 1:** Characteristics of manitoba CLL population

	Referred patients	Nonreferred patients
*N* (%)	281 (51)	274 (49)
Median age (range)	68 y (39–99)	72 y (45–96)
Male to female ratio	1.8:1	1.8:1
SLL (%)	46 (16)	34 (12)
Treated for CLL (%)	77 (27)	31 (11)
Treated for other cancers (%)	10 (3.6)	11 (4.0)
Other cancers (%)	Pre‐CLL: 33 (12)	Pre‐CLL: 40 (15)
	Concurrent: 7 (2.5)	Concurrent: 11 (4)
	Post‐CLL: 15 (5)	Post‐CLL: 13 (5)
Median time to 2nd cancer (Range)	24 m (2–48)	15.6 m (2.5–52.8)
Urban to rural ratio	1.1:1	1.4:1
Mortality (%)	28 (10)	63 (23)

CLL, chronic lymphocytic leukemia; m, month; SLL, small lymphocytic lymphoma; TTFT, time to first treatment; y, year.

**Table 2:** Causes of death in referred and nonreferred patients

Causes of death	Referred patients (%)	Nonreferred patients (%)
CLL/SLL	16 (57)	22 (35)
CLL complications^a^	1 (4)	2 (3)
Other hematopoietic malignancies^b^	2 (7)	6 (9.5)
Solid tumors^c^	4 (14)	10 (16)
Nonmalignant^d^	2 (7)	6 (9.5)
Unknown	3 (11)	17 (27)
Total	28 (100)	63 (100)

a Large B‐cell lymphoma (1) in referred patients and infection (1) and immune thrombocytopenic purpura (1) in nonreferred patients.

b Multiple myeloma (1) in referred patients and acute myeloid leukemia (2) and multiple myeloma (4) in nonreferred patients.

c Lung (1), nonmelanoma skin cancer (1), endometrium (1) and prostate (1) in referred and digestive organs (2), lung (3), brain (2), and unspecified site (3) in nonreferred patients.

d Heart disease (2) in referred and diabetes (2), heart disease (2), stroke (2), respiratory disease (1), and muscular disorder (1) in nonreferred patients.

### Univariable analysis

**Figure 2:**
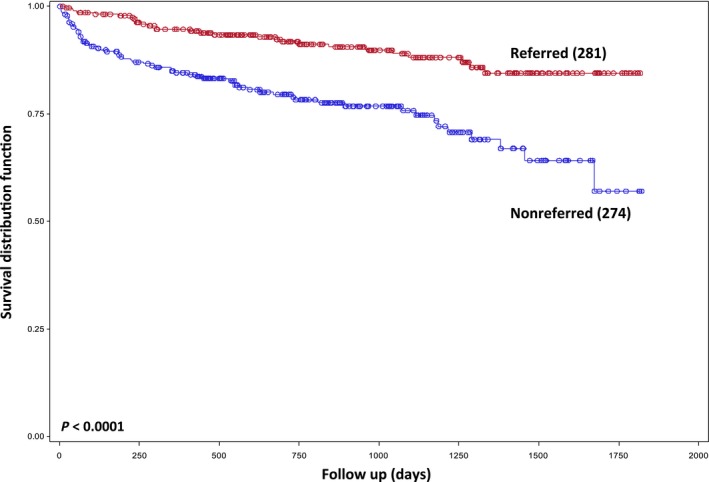
Kaplan–Meier plot of overall survival based on referral status.

**Figure 3:**
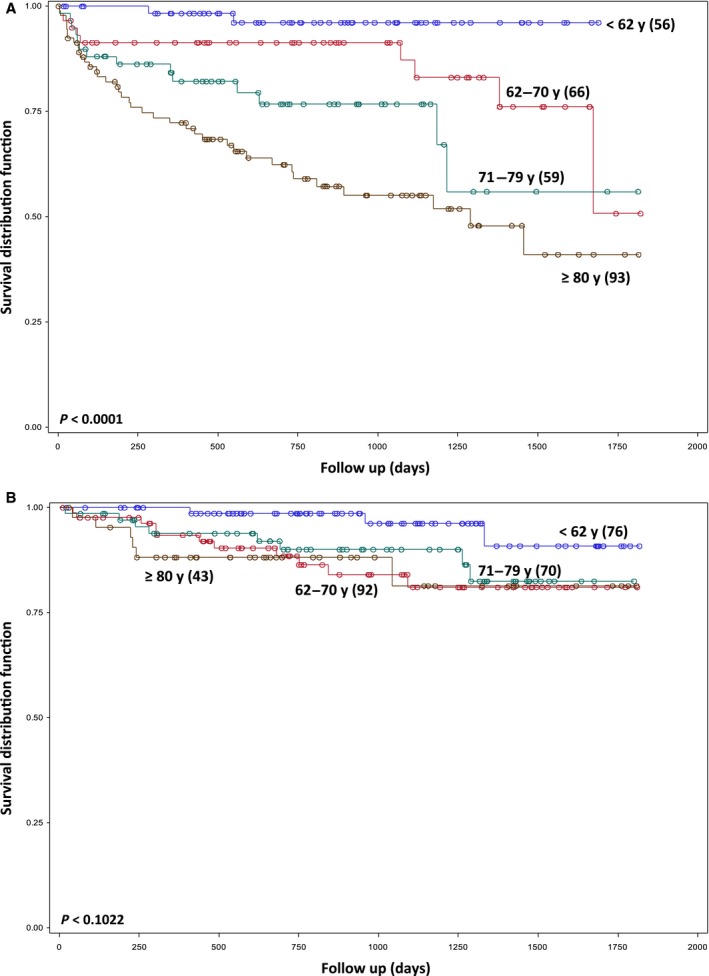
Kaplan–Meier plot of overall survival based on age in nonreferred (A) and referred (B) patients; y: year; (*n*).

Patients that were not referred to the CLL clinic had an increased risk of mortality compared with referred patients (HR 2.74, *P* < 0.0001) (Fig. 2, Table 3). In referred patients, age ≥62 years, requiring treatment for CLL and having a previously diagnosed cancer were associated with poor OS. In contrast, in nonreferred patients, OS continued to decline with advancing age (Fig. 3, Table 3). In this group, presence of a previously diagnosed cancer and requiring treatment for other malignancies were also associated with poor survival. As opposed to referred patients, treatment for CLL was not associated with outcome in nonreferred patients (Table 3).

**Table 3:** Univariable analysis of overall survival

Univariable analyses (overall survival)			
Variable	All CLL patients HR (95% CI)	Referred patients HR (95% CI)	Nonreferred patients HR (95% CI)
Referral status			
Referred	1.00	–	–
Nonreferred	2.74 (1.75–4.28)^a^		
Age quartile			
<62 yr	1.00	1.00	1.00
62–70 yr	4.34 (1.63–11.55)^a^	4.04 (1.13–14.49)^a^	4.73 (1.02–21.88)^a^
71–79 yr	5.49 (2.08–14.50)^a^	3.20 (0.85–12.06)	8.94 (2.03–39.33)^a^
≥80	11.83 (4.70–29.85)^a^	4.56 (1.14–18.26)^a^	15.72 (3.79–65.14)^a^
Gender			
Female	1.00	1.00	1.00
Male	1.37 (0.88–2.14)	1.50 (0.66–3.41)	1.32 (0.78–2.25)
Tx‐CLL			
Untreated	1.00	1.00	1.00
Treated	1.18 (0.73–1.90)	2.19 (1.04–4.60)^a^	1.12 (0.55–2.28)
Tx‐other cancers			
Untreated	1.00	1.00	1.00
Treated	2.27 (1.05–4.91)^a^	1.94 (0.46–8.17)	2.59 (1.04–6.50)^a^
Pre‐CLL cancer			
Without	1.00	1.00	1.00
With	2.34 (1.42–3.87)^a^	2.80 (1.13–6.96)^a^	2.03 (1.11–3.70)^a^
Post‐CLL cancer			
Without	1.00	1.00	1.00
With	1.72 (0.86–3.42)	2.30 (0.80–6.64)	1.56 (0.63–3.89)
Residence			
Urban	1.00	1.00	1.00
Rural	1.03 (0.68–1.56)	1.21 (0.58–2.55)	1.02 (0.62–1.70)

CI, confidence interval; CLL, chronic lymphocytic leukemia; HR, hazard ratio (estimated for mortality); Tx, treatment; yr, year.

a *P* < 0.05.

### Multivariable analysis

When adjusted for age, gender, treatment, pre‐ and post‐CLL cancers and residency in a multivariable model, nonreferral to the CLL clinic was strongly associated with increased mortality (HR 2.39, *P*: 0.0005). In referred patients, being treated for CLL, pre‐CLL cancer and advancing age were associated with decreased OS; in contrast, in nonreferred patients, only age was significantly associated with OS (Table 4).

**Table 4:** Multivariable analysis of overall survival

Multivariable analysis (overall survival)			
Variable	All CLL patients HR (95% CI)	Referred patients HR (95% CI)	Nonreferred patients HR (95% CI)
Referral status			
Referred	1.00	–	–
Nonreferred	2.39 (1.5–3.82)^a^		
Age quartile			
<62 yr	1.00	1.00	1.00
62–70 yr	4.07 (1.52–10.86)^a^	4.33 (1.19–15.70)^a^	4.21 (0.91–19.54)
71–79 yr	5.44 (2.04–14.49)^a^	3.12 (0.80–12.28)	8.57 (1.94–37.87)^a^
≥80	10.76 (4.19–27.58)^a^	5.01 (1.16–21.68)^a^	16.38 (3.93–68.26)^a^
Gender			
Female	1.00	1.00	1.00
Male	1.61 (1.02–2.55)^a^	1.57 (0.67–3.65)	1.64 (0.95–2.84)
Tx‐CLL			
Untreated	1.00	1.00	1.00
Treated	1.58 (0.96–2.60)	2.32 (1.06–5.08)^a^	1.16 (0.56–2.39)
Pre‐CLL cancer			
Without	1.00	1.00	1.00
With	1.85 (1.11–3.10)^a^	2.88 (1.07–7.74)^a^	1.78 (0.96–3.29)
Post‐CLL cancer			
Without	1.00	1.00	1.00
With	2.31 (1.14–4.70)^a^	2.66 (0.86–8.26)	1.98 (0.77–5.07)
Residence			
Rural	1.00	1.00	1.00
Urban	1.16 (0.76–1.77)	1.21 (0.56–2.59)	1.18 (0.50–3.18)

CI, confidence interval; CLL, chronic lymphocytic leukemia; HR, hazard ratio (estimated for mortality); Tx: treatment; yr, year.

a *P* < 0.05.

## Conclusions

Previous studies have suggested that the outcome of cancer patients is influenced by where they receive their care and treatment. Thus, outcome appears to be improved when caregivers have a specific interest in the disease in question and where large numbers of patients with the disease are managed. It has been suggested that this is related to the cumulative expertise of support staff, better access to new agents through clinical trials and the better monitoring and management of comorbidities, toxicities and disease‐specific complications 6, 7, 8, 10, 12. Moreover, these centers are more likely to develop treatment guidelines and to practice evidence‐based medicine, factors that are associated with improved patient outcome 13, 14. In the case of CLL, patients seen in a CLL clinic are more likely to be evaluated using molecular prognostic markers, to participate in clinical trials and to receive purine nucleoside‐based therapy, rather than single alkylating agents or monoclonal antibodies 10.

Population studies show that the median age at diagnosis for CLL patients is 72 years 1, while in clinics the median age at diagnosis is much younger (64 years at the Mayo Clinic and 58 years at MD Anderson Cancer Center) 15, 16. In this study, the median age at diagnosis for referred patients was 68 years with a relatively normal age distribution, while nonreferred patients had a bimodal age distribution with two equal age peaks at 60 and 80 years. In addition, while the median age of the CLL clinic is relatively close to the population median, there are still a substantial number of elderly patients in the nonreferred group indicating referral bias. This suggests that younger patients are being referred to the clinic for therapy, while elderly patients may not be referred as they are not considered fit enough to receive treatment. This would explain why the relative survival of elderly CLL patients has not changed over the past 20 years 4. While it might be expected that distance from the clinic would also influence referral practice, this did not appear to be a factor in our study.

Our findings would suggest that elderly patients are the primary beneficiaries of attending the CLL clinic. Thus, while patients, aged ≥62 years, had a poorer prognosis than younger patients in the clinic, survival did not decrease further with advancing age. In contrast, there was a continued decrease in survival with age in nonreferred patients. We also observed that while mortality due to comorbidities was similar in both cohorts there were more CLL deaths attributed to CLL or its complications in nonreferred patients. This difference in survival between the two cohorts may be related to the fact that older patients in the clinic were more likely to receive chemotherapy than nonreferred patients. Alternatively, this may reflect referral bias, as elderly patients with comorbidities may not have been referred to the CLL clinic. However, the improved survival in the CLL clinic could also have been related to improved therapy and supportive care. Patient survival has improved in the past 15 years with the development of fludarabine and rituximab 2, 17. The addition of rituximab markedly improves the effectiveness of chemotherapy; however, rituximab must be infused in a supervised setting, and nonreferred patients may thus be less likely to receive chemoimmunotherapy than referred patients.

Shanafelt et al. 10 have shown that the survival of CLL patients seen at the Mayo Clinic was longer than patients in the Surveillance, Epidemiology and End Results (SEER) registry. Although this observation could also be attributed to referral bias, they also demonstrated a longer TTFT and OS for CLL patients seen by a hematologist specializing in CLL at the Mayo Clinic as compared to patients seen by other hematologists at the same center. Furthermore, outcome of patients referred to the Mayo Clinic and primarily cared for by a fellow was determined based on whether the supervising physician was a CLL hematologist or a non‐CLL hematologist. Thus, physician disease‐specific expertise was an independent and important prognostic factor for CLL patients and referral bias did not explain the improved survival for patients treated by CLL specialists.

A UK study also reported that early stage CLL patients that were evaluated by hematologists at two large hospitals and were discharged to the primary care had a similar outcome to early stage patients that were followed by the hematologists at the hospital 18. However, it should be emphasized that only early stage patients with stable white blood count were approved for follow up by primary care and these patients were re‐referred to the hematologists once they showed the first sign of disease progression. Therefore, both groups, whether followed by hematologists or primary care, equally benefited from specialist expertise in the management of their disease.

Contrary to our previous report which evaluated patients diagnosed between 1998 and 2003 1, in this study, we observed that gender did not influence survival in the present cohort, evaluating patients from 2007 to 2011. The improved survival of male patients observed in this study may be attributed to the more effective treatments available today. In the previous study, most patients received single‐agent chlorambucil or fludarabine, whereas in the latter cohort, most patients received rituximab with a fludarabine‐containing regimen. Other investigators have also observed improved survival of male patients, as compared to females, over the past decade 3, 4. As a result gender differences in survival has decreased over time.

For our patients in the clinic, a history of a previous cancer and requiring therapy for CLL were associated with a decreased OS. This is consistent with data from the MD Anderson, where having had a prior malignancy was associated with decreased survival 19. A postulated mechanism for this observation is that patients who had received prior chemotherapy or radiotherapy would be less able to tolerate chemotherapy for their CLL. This was less of an issue for nonreferred patients as they were less likely to receive treatment for their CLL.

It has previously been shown that geographic location is as an independent risk factor for survival in patients with lymphoma, with rural patients having an inferior outcome regardless of prognostic indicators 14. However, in our study, place of residence did not influence survival in CLL, either for referred or nonreferred patients. This is likely because cancer patients in rural Manitoba are managed through local cancer clinics with treatment being overseen by the patient's hematologist at CCMB.

Despite our findings, in this study, we were expecting survival to be higher in the nonreferred cohort as patients with more benign disease would be less likely to be referred. Moreover, it was thought likely that a number of the nonreferred patients had MBL, whereas patients in the CLL clinic with MBL (15% of the population) were excluded from the analysis. Considering the premalignant nature of MBL, inclusion of these patients in the nonreferred population should have improved survival in this group as compared to the referred patients. Moreover, some patients had been initially followed by general hematologists and family physicians but referred to the CLL clinic with disease progression. These patients were included in referred cohort and therefore there are some crossovers between the two groups. Unfortunately data pertaining Rai staging, comorbidities and type of treatment were not available for this study.

In summary, this is the first study in Canada exploring the outcome of CLL patients who were either referred or not referred to a CLL specific clinic. Patients seen in the specialized clinic tended to be younger than nonreferred patients, but correcting for age and gender, the OS was substantially higher for patients seen in the specialized clinic. This finding could be attributed to the marked improvement in survival of elderly patients referred to the CLL clinic, who were more likely to receive chemotherapy than nonreferred patients. The increased likelihood of elderly referred patients receiving therapy may be related to a difference in treatment practices in the CLL clinic and the community. Alternatively, there may have been referral bias with elderly CLL patients not being referred to the CLL clinic if they had significant other comorbidities.

Ongoing studies are examining these possibilities and in particular are assessing why the elderly are not being referred to specialized clinics. These studies are particularly relevant at this time, because of the recent development of novel, nontoxic and highly effective antitumor agents which could be tolerated by elderly patients with CLL.

This study exemplifies the differences between patients seen in referral centers and those in the general population. In addition, it demonstrates that improvements observed in the clinic with the development of new therapies, may not necessarily be enjoyed by the entire population and whether this is a reflection of referral bias or differences in treatment practices remains unclear. This should be explored in further studies on CLL and other diseases.

## Conflict of Interest

JBJ and VB sit on advisory boards for Roche and Lundbeck Pharmaceuticals. JBJ and SBG receive research funding from Roche Pharmaceuticals. This study was not influenced by the funding source.
